# Safety of Immune Checkpoint Inhibitors in Elderly Patients: An Observational Study

**DOI:** 10.3390/curroncol28050283

**Published:** 2021-08-25

**Authors:** Agnese Paderi, Sara Fancelli, Enrico Caliman, Serena Pillozzi, Elisabetta Gambale, Marinella Micol Mela, Laura Doni, Francesca Mazzoni, Lorenzo Antonuzzo

**Affiliations:** 1 Medical Oncology Unit, Careggi University Hospital, 50134 Florence, Italy; agnese.paderi@unifi.it (A.P.); sfancelli.sf@gmail.com (S.F.); enrico.caliman@unifi.it (E.C.); serena.pillozzi@unifi.it (S.P.); gambaleelisabetta@gmail.com (E.G.); micolmela@yahoo.it (M.M.M.); doni.laura@gmail.com (L.D.); mazzonifr@aou-careggi.toscana.it (F.M.); 2 Department of Experimental and Clinical Medicine, University of Florence, 50134 Florence, Italy

**Keywords:** aging, immunosenescence, ICI, elderly, skin toxicity, endocrinologic toxicity, irAEs, PD-1

## Abstract

Background: Immunotherapy has completely changed the treatment of solid tumors. Although immune checkpoint inhibitors (ICIs) seem to be an appealing alternative to chemotherapy, especially in elderly patients, due to a more tolerable toxicity profile, they can lead to a peculiar variety of immune-related adverse events (irAEs). However, data on tolerability and outcome of ICIs in the elderly are lacking due to poor accrual in clinical trials of these patients. Methods: We performed a retro-prospective analysis on patients treated with single agent anti-PD-L1/PD-1 at the Clinical Oncology Unit, Careggi University Hospital, from March 2016 to March 2020. Data on the treatment responses, type and severity of irAEs, as well as the corticosteroids (CCS) dosage used for irAEs and the discontinuation rate, were described per each patient, according to two different age-based cohorts of patients (< or ≥70 years). Results: We reported a lower incidence of all-grade toxicity in elderly compared to younger patients (64.9% vs. 44.9%, *p* = 0.018). The two age-cohorts showed a different profile of irAEs. Endocrine irAEs were significantly higher in younger patients (39.7% vs. 21.7%, *p* = 0.002), while dermatologic toxicities were more common in the older group (35.0% vs. 11.3%, *p* = 0.047). Use of CCS and treatment discontinuation rate do not differ significantly between the two age groups. Conclusion: Our findings suggest that treatment with ICIs in elderly populations is safe and feasible. Patients over 70 years are more prone to develop skin irAEs, while younger patients are more subject to experience endocrine toxicities.

## 1. Introduction

Age is the most important risk factor for many chronic diseases including malignancies [1]. Cancer prevalence and incidence increase with aging [2] and about 50% of new cases worldwide arise in older patients [3]. According to the World Health Organization definition, people over 65 are described as elderly, without considering the progressive increase in life expectancy in Western countries, now estimated at an average of 72 years globally (with maximum peaks in the most virtuous states of 80 years for males and 84 years for females) [4]. However, into the field of geriatric oncology, the age of 70 is the most commonly used cut-off to define elderly patients in clinical trials [5].

Over the last years, the cancer treatment scenario has undergone a complete revolution with the advent of immunotherapy. Immune checkpoint inhibitors (ICIs) are the most substantial novelty of the last years, with wide applicability to both solid and non-solid oncological pathologies, leading to a satisfactory performance in outcome and tolerability. While in hematological pathologies PD-L1 and other factors have prognostic value, in solid malignancies we currently stratify patients by clinical characteristics such as age and comorbidities [6,7]. As the number of elderly treated with ICIs is steadily increasing, data on the outcome and toxicity profile in this sub-population appear critical. Despite the difficulty of enrolling elderly patients in clinical trials, few real-world experiences of elderly patients with non-small cell lung cancer (NSCLC) and renal cell cancer (RCC) treated with ICIs have shown a similar efficacy, without a detrimental effect in tolerance compared to younger patients [8,9,10,11,12,13,14]. Moreover, in a recent metanalysis of six studies on NSCLC that compared ICIs with chemotherapy in the first or subsequent line of therapy, the hazard ratio (HR) for overall survival (OS) was comparable between patients < or ≥75 years (HR = 0.87; 95% CI: 0.56–1.35) [15].

Even though ICIs are usually well-tolerated, compared with traditional chemotherapy (CT), they are associated with a peculiar spectrum of adverse events (AE). Due to their mechanism of action, ICIs can induce inflammatory side effects known as immune-related AE (irAEs), which are unique and different from those of conventional anticancer therapies. Despite their favorable toxicity profile, one of the unsolved goals is the stratification of population through predictive criteria of toxicity, discontinuation and hospitalization, which is easily reproducible in everyday clinical activity, with metrics such as performance status (PS), comorbidity and age. It is not yet established if older patients experience the same amount, severity and subtype of irAEs as the younger patients.

Retrospective data from clinical trials and global databases suggest a different distribution of irAEs between elderly and young patients, with a prevalence of high-grade AEs in elderly with a poorer PS [16]. However, older patients in good conditions treated with ICIs show an equal distribution of toxicity compared to the younger population [17]. Otherwise, other studies suggested that aging could confer a protective effect against irAE development [18,19]. The type and the severity of irAEs may be different in older compared with younger patients, as aging is associated with immunosenescence, which can impair the immune response [20,21,22,23,24].

In this article, we describe the toxicities reported in a group of elderly patients treated with anti-PD-L1/PD-1 with a distinction by type, severity, number and discontinuation rate; we later focus on the treatment of irAEs.

## 2. Materials and Methods

### 2.1. Study Population

We retrospectively collected clinical data from adult patients (age ≥18 years) treated with single agent anti-PD-L1/PD-1, from March 2016 to March 2020 at the Clinical Oncology Unit, Careggi University Hospital, Florence (Italy). We enrolled patients with advanced NSCLC, melanoma and RCC who received pembrolizumab 200 mg three times weekly, nivolumab 3 mg/kg or 240 mg every 2 weeks or 480 mg monthly or atezolizumab 1200 mg every 3 weeks. Exclusion criteria were as follows: lack of consent, age <18 years old, ECOG PS >2, active autoimmune diseases or concomitant use of corticosteroids (CCS) at the time of enrollment (prednisone dose ≥10 mg or equivalent per day). ICIs were administered in the first, second and third lines of therapy in agreement with the Italian regulatory agency (AIFA).

Patients were divided into two groups according to age, between elderly (≥70 years) and younger patients (<70 years). Data about type, duration, grade of toxicity and CCS treatment distinguished by age were described. We also collected data about survival and best response. IrAEs were evaluated according to the Common Terminology Criteria for Adverse Events version 5.0 (CTCAE v5.0—https://ctep.cancer.gov/protocoldevelopment/electronic_applications/docs/ctcae_v5_quick_reference_5x7.pdf, accessed on 14 July 2021), distinguishing them by the organs affected. The best response was determined by analyzing the imaging data (CT scan) in accordance with the iRECIST criteria. PS was described according to the Eastern Cooperative Oncology Group (ECOG).

All data were analyzed anonymously; all patients signed an informed consent form for immunotherapy with particular specifications about possible AE occurrence. This study was conducted in accordance with the World Medical Association Declaration of Helsinki and independently reviewed and approved by the Regional Ethics Committee for Clinical Trials of the Tuscany Region (approval No.: 17332_oss). All patient data were processed in anonymity and de-identified prior to analysis.

### 2.2. Statistical Analysis

Estimates of incidence and subtypes of irAEs, according to different age groups, were collected and statistical comparisons for categorical variables were performed using X^2^ test. Data were analyzed using the statistical software Jamovi [25].

## 3. Results

Data of 146 patients were analyzed according to the two age cohorts (<70 years and ≥70 years). Patient characteristics are summarized in Table 1. In the study population, 89 patients were ≥70 years (60.9%), and the median age was 70 years (range: 27–91 years). Among the elderly patients, most were male (*n* = 62, 69.7%), smokers (*n* = 61, 68.5%) and with PS 0-1 at the time of diagnosis (*n* = 83, 93.2%). NSCLC was the most common tumor in the elderly cohort (*n* = 40, 44.9%), followed by melanoma (*n* = 29, 32.6%) and RCC (*n* = 20, 22.5%). In this group, ICIs were administered predominantly in the second line (49.4%). Among elderly patients, the disease control rate (DCR) was 45% (2 complete response, 14 partial response and 24 stable disease), while disease progression was reported for 49 patients (55%). No differences in DCR were observed between the two study group populations (*p* = 0.265).

Overall, ICIs were well tolerated in both elderly and younger patients (Table 2). We reported a significantly higher incidence of all-grade toxicity in the <70 age group, which appears more susceptible to the occurrence of AE (64.9% vs. 44.9%, in younger and elderly cohorts, respectively; *p* = 0.018). However, no difference was reported between the two age groups regarding the number (*p* = 0.227) and severity (*p* = 0.624) of toxicities. Moreover, the treatment discontinuation rate was similar between the two cohorts (5.3% vs. 4.5%, in younger and older populations, respectively; *p* = 0.832)

**Figure 1:**
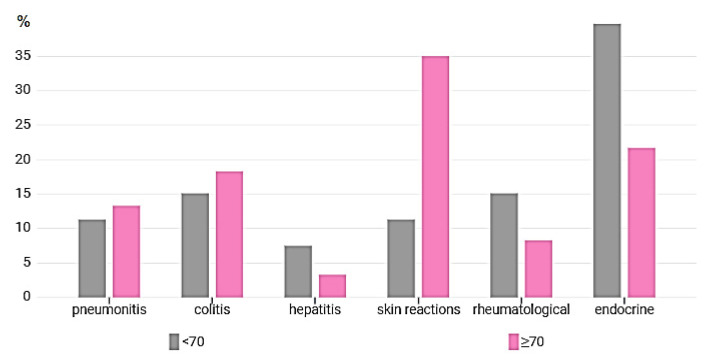
Graphic representation of the distribution of toxicities divided by age groups and type of irAE.

Interestingly, we found a significant difference in the occurrence of irAE subtypes. The endocrine AEs were described more often in young patients than in elderly patients (39.7% vs. 21.7%, respectively; *p* = 0.002), while the dermatologic toxicities were more common in the >70 age group, compared to the young population (35.0% vs. 11.3%, respectively; *p* = 0.047) (Table 3, Figure 1). When we analyzed the toxicities for each histotype in relation to age, despite the small sample size, a similar statistically significant difference was observed in NSCLC (Tables S1–S3).

We then evaluated CCS treatment for irAEs per each age group (Table 4). Overall, of the 77 patients who experienced at least one toxicity, 41 were treated with steroids. No statistically significant differences were observed between the two groups in duration and dosing of CCS. However, a higher percentage of elderly patients received CCS for a longer period (≥2 weeks) compared to the younger group (53.9% vs. 40.0%, respectively).

## 4. Discussion

ICIs have radically changed the current state of treatment in many malignancies and seem to be an appealing alternative to conventional therapy, especially for older subjects, due to a more tolerable toxicity profile. However, ICIs can cause a variety of irAEs [17,26]. The spectrum of ICI-induced toxicities may be different in older patients compared with younger populations, as immunosenescence can dampen the immune system and this may impact the toxicity profile in such sub-populations [20,21,22,23]. As older people are often under-represented from registration trials of ICIs, data regarding toxicities in the elderly are lacking. 

In our study, we retrospectively investigated data from two age-based cohorts of patients, with a cut-off of 70 years. We reported that ICIs were well-tolerated in both older and younger populations. The proportion of patients with overall all-grade toxicity was significantly higher in the younger group of patients (*p* = 0.018). However, there was no significant difference in the distribution of serious (≥G3) and non-serious irAEs between the two groups. A similar result has been reported in a real-world experience where the rate of any-grade irAEs was lowest in patients ≥80 years (59%), and highest in patients <50 years (92%). [27]. A well-tolerated profile of toxicities in the older patient age-groups has been reported in other retrospective analyses [18,28,29] in the Italian expanded access programs of nivolumab in NSCLC and in RCC, in programs of ipilimumab in melanoma [8,13,30] and in the phase 3B/4 checkmate 153 study, assessing nivolumab in patients with advanced NSCLC with poor prognostic features of advanced age or diminished ECOG PS [31].

The impact of immunosenescence on the development of immune-related toxicity in elderly patients is controversial. Aging is a heterogeneous phenomenon due to the result of diet behavior, professional and environmental exposure, physical activity and, significantly, genetic assessment [1]. This complex and multifactorial path generates a progressive decline of metabolic and physiologic cellular turnover due to an imbalance between the increase of reactive oxygen species (ROS) and the reduction of anti-inflammatory scavengers’ accrual [32]. Aging alterations in both arms of the immune system, as well as in their efficient cooperation, contribute to the development and maintenance of inflammaging [31]. The lack of equilibrium between the innate and acquired immune system is seen with the decreased activity of natural killer (NK) cells and the reduction of polymorphonucleate (PMN), macrophage, dendritic cells (DC) and CD4+ T cells [20]. Meanwhile, CD8+ T cells are exhausted and decreased in peripheral blood and they show an increased expression of surface receptors as PD-L1, CTLA-4, LAG-3 and TIM-3, involved in the immune inhibition pathway [21,24]. The depletion of medullary cell storage [33], the progressive thymic involution [34] and the metabolic competition between immune system cells and cancer cells, which both take advantage of the aerobic glycolysis, represent additional causes involved in the genesis of immune imbalance [35,36]. Even though there is no evidence yet of a mechanistic reason explaining a lower incidence of toxicities in elderly, all of the mentioned factors could be integrated into explaining why older patients seem to experience less toxicities compared with younger cohorts.

Interestingly, we also described a difference in the subtype distribution of irAEs between elderly and young patients. We reported that the endocrine toxicity rate was significantly higher in the younger population (39.6%) than in the older group (21.6%) (*p =* 0.002). Our findings are consistent with data described in a previous retrospective multicenter study [37], in which immune-related toxicities were analyzed based on three age cohorts in patients with advanced solid cancers. The authors described a significantly lower incidence of all-grade endocrine toxicity in the oldest cohort (11.0%) compared with the youngest cohort (11.0% vs. 22.7%, respectively, *p* = 0.02; OR 0.43; 95% CI 0.21–0.87). However, a clear physiopathological explanation for the higher rate of endocrine AEs in the elderly population is lacking. A similar trend toward a higher rate, although not statistically significant, was shown for rheumatologic irAEs in our study: 15.1% patients of the younger cohort and 8.3% of the elderly cohort, respectively, experienced rheumatologic toxicities (*p* = 0.081). Conversely, a significant increase of dermatological toxicity was reported in the older cohort, compared to the young population (35% vs. 11.3%, respectively, *p =* 0.047). According to the latter result, in the analysis by Singh et al. [38], in which the safety of nivolumab was described by age for patients enrolled in phase 3 registration trials, specific irAEs were similar according to the different organs in each age group, except for diarrhea and dermatological toxicity, which were more present in elderly groups. Intriguingly, immunosenescence has been linked to a proinflammatory state of the skin that may contribute to the high frequency of inflammatory skin reactions in older patients [39]. We can argue that this proinflammatory condition predisposes elderly treated with ICIs to develop immune-related skin toxicity more frequently compared to younger patients.

Consistently with our results, another meta-analysis, which reported clinical data from 21 phase II/III ICIs trials based on age stratification into young and old subgroups, has described an overall profile of low toxicity, both in younger and older patients treated with immunotherapy [40]. Even when high-grade irAEs were reported in our study, the duration and dosing of CCS treatment was similar throughout the age groups, and the discontinuation rate due to toxicities was not significant between the two sub-populations. Thus, our findings together with previously published data, correlating age with immunotherapy, suggest that ICIs are well-tolerated in the elderly, while showing a different profile of irAE subtypes.

In our study, the efficacy of immunotherapy was similar between the two age cohorts (DCR 49% vs. 48%, in younger and elderly groups, respectively; OR = 1.05, 95% CI: 0.54–2.01; *p* = 0.265). These findings are consistent with data from two meta-analyses, in which several clinical trials testing ICIs in different cancer types, including melanoma, RCC and NSCLC, were evaluated [40,41]. The effectiveness of ICIs in elderly patients has been confirmed also in data from real-life experiences and global databases [8,9,10,11,14,42]. In RCC, several clinical trials and the Italian EAP exploring the efficacy and tolerability of ICIs, have demonstrated an effect in patients over 75 [13,43].

Our paper clearly has some limitations, mainly the definition of the two cohorts of patients based only on an age cut-off and the tumor heterogeneity. Moreover, although the incidence of irAEs was not higher in the elderly population, it could be argued that their impact on clinical outcomes in these patients may be more pronounced due to their frailty and co-morbidities. Despite its shortcomings, our paper could deliver an important message to clinical oncologists stressing the importance of treating patients with ICIs regardless of their age. At the same time, clinicians should be particularly aware of the different toxicity profile in elderly and young patients.

Further assessments for safety and efficacy of ICIs in the elderly are warranted in prospective trials, considering the higher incidence of cancers among this setting of patients. It would be necessary to expand the enrollment of elderly population in prospective studies in order to define the weight of the so-called immunosenescence on the outcome of ICI treatments. An increased awareness of molecular and metabolic factors could increase the knowledge about mechanisms linked to aging.

## Figures and Tables

**Table 1:** Patient characteristics.

Patients’ Characteristics	Overall *n* = 146 (%)	<70 *n* = 57 (%)	≥70 *n* = 89 (%)	*p* (<70 vs. ≥70)
Sex				
Male	98 (67.1)	36 (63.2)	62 (69.7)	*p* = 0.414
Female	48 (32.9)	21 (36.8)	27 (30.3)	
Age, years				
Average	67.5	55.2	75.4	
Median	70	57	75	
Min–Max	27–91	27–69	70–91	
Smoker				
Yes	99 (67.8)	38 (66.6)	61 (68.5)	*p* = 0.813
No	47 (32.2)	19 (33.4)	28 (31.4)	
PS at the time of diagnosis				
0	100 (68.5)	44 (77.1)	56 (62.9)	*p* = 0.190
1	38 (26.0)	11 (19.2)	27 (30.3)	
2	8 (5.5)	2 (3.5)	6 (6.7)	
Primary site				
NSCLC	67 (45.9)	27 (47.4)	40 (44.9)	*p* = 0.937
Melanoma	46 (31.5)	17 (29.8)	29 (32.6)	
RCC	33 (22.6)	13 (22.8)	20 (22.5)	
Therapy line				
First	63 (43.2)	25 (43.8)	38 (42.7)	*p* = 0.822
Second	70 (47.9)	26 (45.6)	44 (49.4)	
Third	13 (8.9)	6 (10.5)	7 (7.9)	
Outcome				
CR	9 (6.2)	7 (12.3)	2 (2.3)	*p* = 0.045
PR	19 (13.0)	5 (8.8)	14 (15.7)	
SD	43 (29.4)	19 (33.3)	24 (27.0)	
PD	75 (51.4)	26 (45.6)	49 (55.0)	

PS = performance status; NSCLC = non-small cell lung cancer; RCC = renal cell cancer; CR = complete response; PR = partial response; SD = stable disease; PD = progressive disease.

**Table 2:** irAE patient description. Events and grading were split into age groups (<70 and ≥70).

	Overall (%) *n* = 146	<70 (%) *n* = 57	≥70 (%) *n* = 89	*p* (<70 vs. ≥70)
Patients who experience toxicity				
yes	77 (52.7)	37 (64.9)	40 (44.9)	*p* = 0.018
no	69 (47.3)	20 (35.1)	49 (55.1)	
number of irAEs /patients				
1	47 (32.2)	20 (54.0)	27 (67.5)	*p* = 0.227
≥2	30 (20.5)	17 (46.0)	13 (32.5)	
max grade (CTCAE) of toxicity/patients				
grade 1–2	50 (34.2)	23 (62.2)	27 (67.5)	*p* = 0.624
grade 3–4	27 (18.5)	14 (37.8)	13 (32.5)	
discontinuation due to an irAE				
yes	7 (4.8)	3 (5.3)	4 (4.5)	*p* = 0.832
no	7 (95.2)	54 (94.7)	85 (95.5)	

CTCAE = Common Terminology Criteria for Adverse Events; irAEs = immune-related adverse events.

**Table 3:** irAE characteristics described by site/organ per each age group.

Type of irAE	All Toxicity *n* = 113 (%)	<70 *n* = 53 (%)	≥70 *n* = 60 (%)	*p* (<70 vs. ≥70)
Pneumonitis	14 (9.6)	6 (11.3)	8 (13.3)	*p* = 0.758
Colitis	19 (13)	8 (15.1)	11 (18.3)	*p* = 0.769
Hepatitis	6 (4.1)	4 (7.5)	2 (3.3)	*p* = 0.157
Skin reactions	27 (18.5)	6 (11.3)	21 (35.0)	*p* = 0.047
Rheumatological	13 (8.9)	8 (15.1)	5 (8.3)	*p* = 0.081
Endocrine related	34 (23.3)	21 (39.7)	13 (21.7)	*p* = 0.002

**Table 4:** Corticosteroids and irAEs. CCS = corticosteroids.

Use of CCS	Overall (%) *n* = 41		*p* (<70 vs. ≥70)
	<70 (%) *n* = 15	≥70 (%) *n* = 26	*p* = 0.704
Duration			
<2weeks	9 (60.0)	12 (46.1)	*p* = 0.393
≥2weeks	6 (40.0)	14 (53,9)	
Dose			
<1mg/kg	8 (53.3)	17 (65.4)	*p* = 0.446
≥1mg/kg	7 (46.7)	9 (34.6)	

## Data Availability

Data are available on request to the corresponding author.

## Supplementary Materials

The following are available online at https://www.mdpi.com/article/10.3390/curroncol28050283/s1, Table S1. Incidence of irAEs in NSCLC population; Table S2. Incidence of irAEs in melanoma population; Table S3. Incidence of irAEs in RCC population.
