# Supplementary material for: Safety of Immune Checkpoint Inhibitors in Elderly Patients: An Observational Study
Source: Curr Oncol. 2021 Aug 25;28(5):3259–67. doi: 10.3390/curroncol28050283 (PMC8395507; doi:10.3390/curroncol28050283)
Supplement: Supplementary file 1 [file curroncol-28-00283-s001.zip › curroncol-1301242-supplementary.pdf]

Current Oncology

MDPI

Safety of Immune Checkpoint Inhibitors in Elderly Patients: An Observational Study

Agnese Paderi [1,†], Sara Fancelli [1,†], Enrico Caliman [1,2], Serena Pillozzi [1], Elisabetta Gambale [1], Marinella Micol Mela [1], Laura Doni [1], Francesca Mazzoni [1] and Lorenzo Antonuzzo [1,2,*]

Supplementary material

Table S1. Incidence of irAEs in NSCLC population.

	**NSCLC n=67 (%)**	**<70 n=27 (%)**	**≥70 n=40 (%)**	**p (<70 vs ≥70)**
**Type of irAEs**	**irAes n = 39**	**irAEs n = 10**	**irAEs n = 19**	
Pneumonitis	6 (9.0)	4 (14.8)	2 (5.0)	*p=0.168*
Colitis	6 (9.0)	2 (7.4)	4 (10.0)	*p=0.715*
Hepatitis	1 (1.5)	1 (3.7)	0 (0.0)	*p=0.220*
Skin reactions	11 (16.4)	1 (3.7)	10 (25.0)	***p=0.021***
Rheumatological	5 (7.5)	2 (7.4)	3 (7.5)	*p=0.989*
Endocrine related	12 (17.9)	8 (29.6)	4 (10.0)	***p=0.040***

Table S2. Incidence of irAEs in melanoma population.

	**melanoma n=46 (%)**	**<70 n=17 (%)**	**≥70 n=29 (%)**	**p (<70 vs ≥70)**
**Type of irAEs**	**irAes n = 32**	**irAes n = 18**	**irAes n = 14**	
Pneumonitis	4 (8.7)	2 (11.8)	2 (6.9)	*p=0.572*
Colitis	8 (17.4)	4 (23.5)	4 (13.8)	*p=0.400*
Hepatitis	1 (2.2)	1 (5.9)	0 (0.0)	*p=0.187*
Skin reactions	7 (15.2)	2 (11.8)	5 (17.2)	*p=0.618*
Rheumatological	4 (8.7)	3 (17.6)	1 (3.4)	*p=0.099*
Endocrine related	8 (17.4)	6 (35.3)	2 (6.9)	***p=0.014***

Table S3. Incidence of irAEs in RCC population.

	**RCC n= 33 (%)**	**<70 n=13 (%)**	**≥70 n=20 (%)**	**p (<70 vs ≥70)**
**Type of irAEs**	**irAes n = 40**	**irAes n = 17**	**irAes n = 23**	
Pneumonitis	4 (12.1)	0 (0.0)	4 (20.0)	*p=0.085*
Colitis	5 (15.5)	2 (15.4)	3 (15.0)	*p=0.976*
Hepatitis	4 (12.1)	2 (15.4)	2 (10.0)	*p=0.643*
Skin reactions	9 (27.3)	3 (23.1)	6 (30.0)	*p=0.663*
Rheumatological	4 (12.1)	3 (23.1)	1 (5.0)	*p=0.120*
Endocrine related	14 (42.4)	7 (53.8)	7 (35.0)	*p=0.284*
